# Plasma Levels of Kynurenines and B‐vitamins across Diagnostic Phases of Dementia and in Healthy Controls

**DOI:** 10.1002/alz70856_102653

**Published:** 2025-12-25

**Authors:** Lieke Bakker, Sebastian Köhler, Kyonghwan Choe, Daniel L.A. van den Hove, Gunter Kenis, Simone J.P.M. Eussen, Miranda T. Schram, Robert J. van Oostenbrugge, Julie Staals, Bart P.F. Rutten, Arve Ulvik, Per M. Ueland, Frans R.J. Verhey, Inez H.G.B. Ramakers

**Affiliations:** ^1^ Alzheimer Center Limburg, Maastricht University, Maastricht, Limburg, Netherlands; ^2^ Mental Health and Neuroscience Research Institute (MHeNs) Maastricht University, Maastricht, Limburg, Netherlands; ^3^ European Graduate School of Neuroscience (EURON), Maastricht University, Maastricht, Limburg, Netherlands; ^4^ Care and Public Health Research Institute (CAPHRI), Maastricht University, Maastricht, Limburg, Netherlands; ^5^ Cardiovascular Research Institute Maastricht (CARIM), Maastricht University, Maastricht, Limburg, Netherlands; ^6^ Mental Health and Neuroscience Research Institute, Maastricht University, Maastricht, Netherlands; ^7^ Heart and Vascular Centre, Maastricht University Medical Centre, Maastricht, Netherlands; ^8^ Department of Internal Medicine, Cardiovascular Research Insitute CARIM, Maastricht University, Maastricht, Netherlands; ^9^ BEVITAL AS, Bergen, Vestland, Norway

## Abstract

**Background:**

The tryptophan‐kynurenine pathway may play a role in cognitive decline in both neurodegenerative and cerebrovascular disorders. However, clinical studies investigating kynurenines so far have generally been small, and studies looking into prodromal disease stages are scarce. The present study used a transdiagnostic approach to investigate levels of kynurenines and B‐vitamins, as important cofactors, across diagnostic phases of dementia and healthy controls.

**Method:**

Blood plasma levels of tryptophan, seven kynurenine metabolites (kynurenine, 3‐hydroxykynurenine, kynurenic acid, xanthurenic acid, anthranilic acid, 3‐hydroxyanthranilic acid, quinolinic acid) and B‐vitamins (pyridoxal 5’‐phosphate and riboflavin) were determined in 2,452 cognitively healthy older adults (used as controls) from the population‐based The Maastricht Study, 759 patients from a memory clinic, including patients with subjective cognitive decline (SCD, n = 293), mild cognitive impairment (MCI, n = 304) or dementia (*n* = 162, and in 194 patients 3 months after stroke with (PSCI, n = 127) or without post‐stroke cognitive impairment (PSNCI, n = 67). Samples were analyzed by Bevital, Bergen, Norway, by means of LC‐MS/MS. Linear regression analyses were done to assess differences between cohorts and across diagnostic phases, while adjusting for a priori defined confounders (demographics, kidney function, and lifestyle factors). Where appropriate, metabolites were log‐transformed prior to analysis and all were standardized for reasons of comparison.

**Result:**

Preliminary results showed that, in analyses comparing stroke patients to controls, plasma levels of tryptophan (‐0.36 [‐0.52, ‐0.21]), xanthurenic acid (‐0.15 [‐0.26, ‐0.00]), kynurenic acid‐to‐quinolinic acid ratio (‐0.45 [‐0.60, ‐0.29]), pyridoxal 5’‐phosphate (‐0.69 [‐0.84, ‐0.54]), and riboflavin (‐0.43 [‐0.58, ‐0.27]) were significantly lower, while levels of kynurenine (0.28 [0.15, 0.41]), 3‐hydroxykynurenine (0.70 [0.56, 0.84]), 3‐hydroxyanthranilic acid (0.45 [0.30, 0.59]), quinolinic acid (0.46 [0.33, 0.60]), and kynurenine‐to‐tryptophan ratio (0.53 [0.40, 0.66]) were higher. No difference were found in kynurenic acid (0.00 [‐0.23, 0.13]) or anthranilic acid (0.01 [‐0.14, 0.16]). Results were similar for patients with PSCI and PSNCI, with no significant differences between these groups. Analyses in the memory clinic population are currently being performed.

**Conclusion:**

These preliminary results suggest that the kynurenine pathway is dysregulated after stroke, independent of cognitive impairment. Further studies are needed to validate these findings.

